# Case Report: Accuracy analysis of a new scanning body for intraoral digital impressions in full-arch edentulous patients

**DOI:** 10.3389/froh.2025.1528943

**Published:** 2025-01-29

**Authors:** Hongyang Ma, Jia Cao, Zhihui Tang, Yuwei Wu

**Affiliations:** The Second Dental Center, Peking University Hospital of Stomatology, Beijing, China

**Keywords:** edentulous, intraoral scanning (IOS), scanning rod, accuracy comparison, digital treatment

## Abstract

This clinical report aimed to compare the scanning accuracy of SRA, and a novel extended scan body in edentulous patients. Through quantitative analysis, the study provided data support for oral prosthodontics and digital treatments. Edentulous patients with six Straumann bone-level implants were selected. The scan data was compared to the standard model derived from traditional impressions to evaluate scanning accuracy. The SRA scan bodies showed lower accuracy (46 ± 45 μm), while the Segma scan bodies with an extended structure achieved significantly higher accuracy (20 ± 2 μm). The extended structure effectively reduced image stitching errors in challenging intraoral regions, improving accuracy and speed. The Segma scan bodies with an extended structure accurately replicated spatial position information of implants in edentulous patients, providing insights for oral prosthodontics and digital treatments to enhance restoration quality.

## Introduction

Patients with dentition loss not only suffer from a near-complete loss of chewing function but also have partial impairments in pronunciation and aesthetics, causing significant physical and psychological impacts (1). The fixed denture restoration supported by multiple implants has effectively improved the retention and stability of traditional complete dentures, greatly enhancing patients' chewing function and quality of life (2). The use of implant-supported fixed denture restoration in the treatment of patients with dentition loss has been proven to be a reliable therapeutic method (3). However, how to accurately obtain the impression of multiple implants remains a major clinical challenge as accurate impression-taking is crucial for the success of these prostheses.

Traditional impression techniques are often associated with limitations in precision and reproducibility (4). With the advancement in digital dentistry, digital impression methods leading implant impression-taking become more precise and efficient, have gained popularity due to their enhanced accuracy and patient comfort (5). However, challenges persist in achieving optimal accuracy, especially in edentulous jaws (6).

The digital impression acquisition for patients with edentulous jaws receiving implant-supported fixed restoration using intraoral scanning technology has always been a research hotspot (7). Traditional scanning bodies are composed of three parts: scanning area, scanning body, and base, which are mostly cylindrical, conical, or spherical with no extended structure. When performing intraoral scanning between multiple implants or implants with a large span in edentulous jaws, the image stitching error is significant, and it cannot provide clinically satisfactory results (8, 9).

This study aims to evaluate the precision of a novel scanning rod in facilitating implant impression-taking for fixed prosthodontic restoration in edentulous patients and provide precise data support for oral restoration and digital treatment by quantitatively assessing the scanning precision of different scanning methods in various intraoral regions. By comparatively analyzing the scanning accuracy of the Straumann screw-retained abutment (SRA) scanning rod, and a novel scanning rod with an extended structure in edentulous patients.

## Clinical report

**Figure 1:**
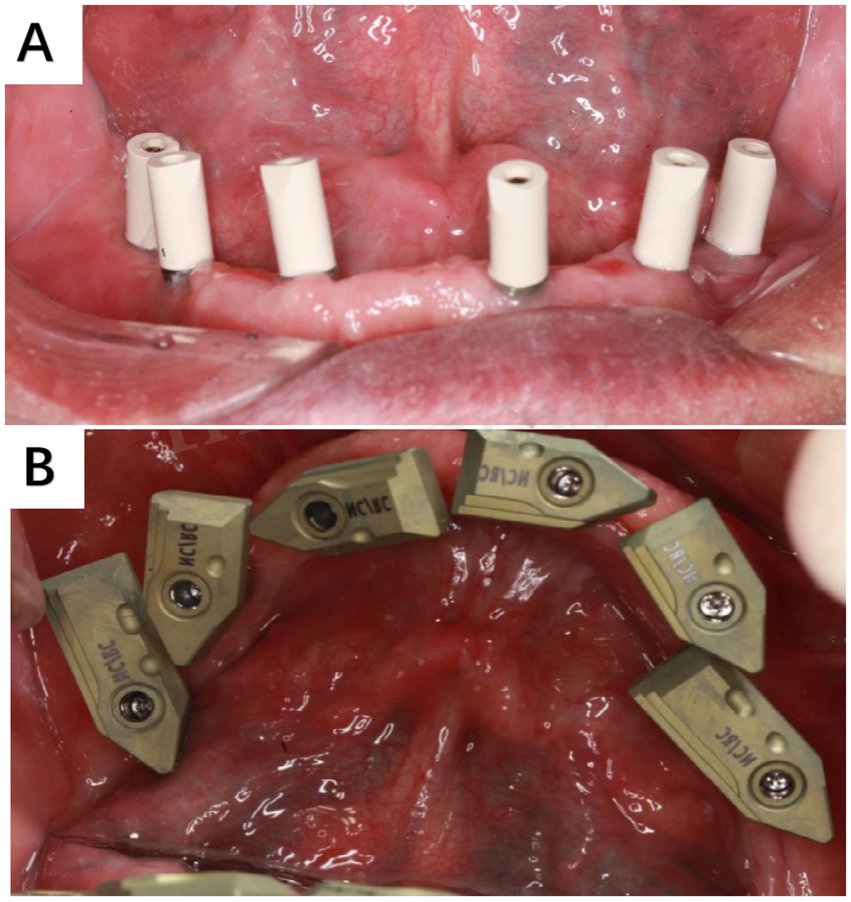
Two different intraoral scan bodies. **(A)** SRA scan procedure; **(B)** Segma scan procedure.

Edentulous patients with six Straumann bone-level implants (Institut Straumann AG, Basel, Switzerland) exhibiting osseointegration in the mandible were selected as the subjects for this study. Traditional impression techniques were initially employed to obtain a mold, followed by intraoral scanning using the CEREC Primescan intraoral scanner (Sirona, Bensheim, Germany) with the SRA scanning rod, and the Segma scanning rod (Digital wings, Segma Medical Tec Co. China) (Figure 1).

**Figure 2:**
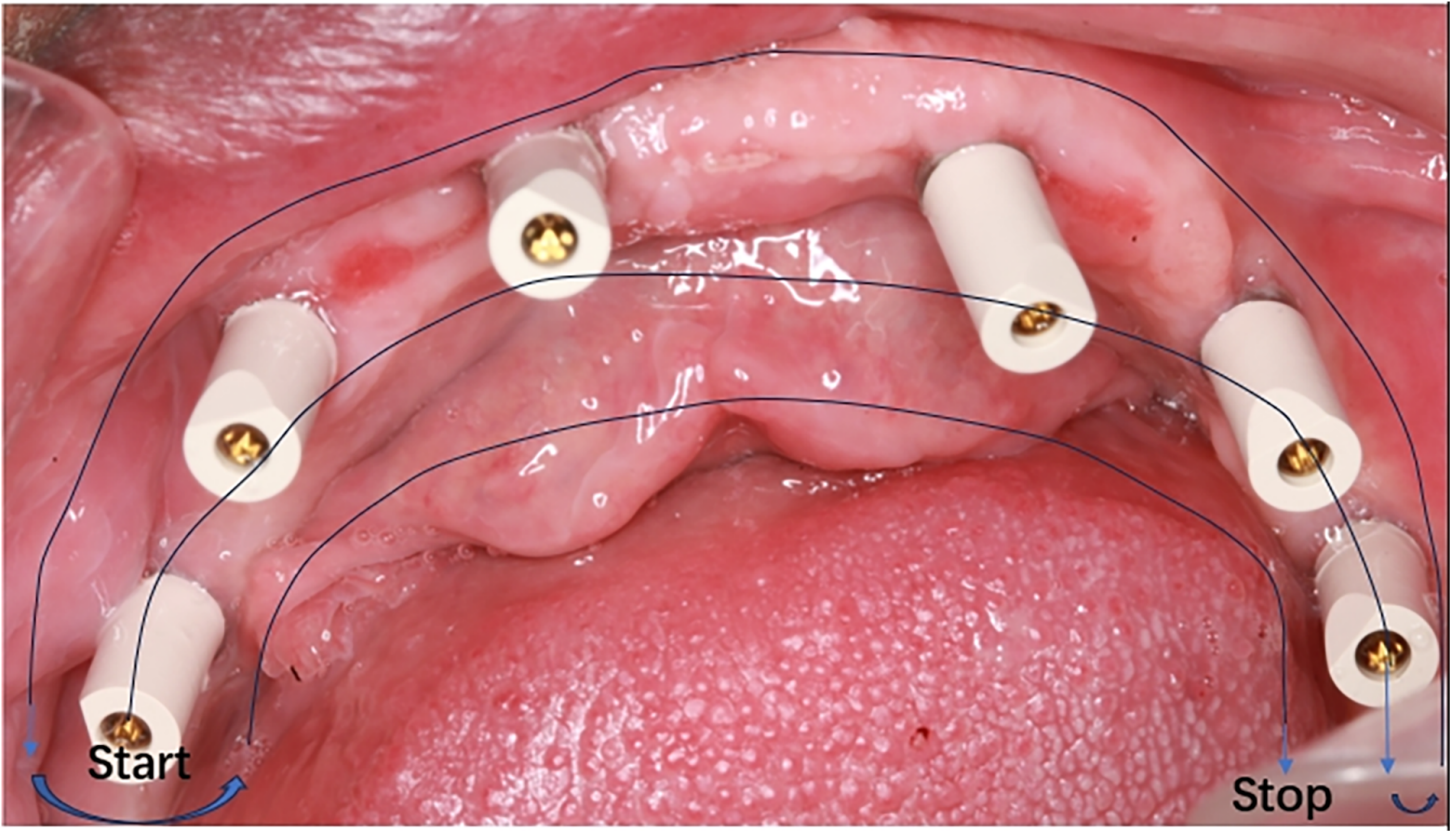
The intra oral scanning strategy.

The scan strategy is as follows: The Primescan Reference Denture scan strategy occurs extra orally. Proceed by scanning only the buccal surfaces, borders, occlusal plane and intaglio of the washed denture. On the maxillary denture it is not necessary to scan the palatal surface. To make the buccal bite scan, hold the dentures together with the bite registration and scan the buccal bite both left and right sides to capture the occlusal alignment. It is important to have overlapping data so the sweeps can be stitched. However, when concerning edentulous cases, we firstly scanned the information of the intraoral mucosa of the edentulous jaw patient in the same manner as described above. After that, we placed the extended scanning rods on the composite abutment, confirmed the position and started scanning the area where the rods needed to be aligned, and then scanned the buccal side for buccal scanning rods and mucous membranes, and then finally moved to the lingual side to scan the lingual side for lingual scanning rods and links and mucous membranes (Figure 2).

For edentulous oral scanning require lens calibration about 10 min prior to scanning, which can be done directly with a special calibrator within 1 min. The scanning process is performed in natural light avoiding the bright light from the dental chair. The operator needs to blow-dry the saliva on the mucosal surface in the mouth, and two-person four-handed operation is recommended to avoid unwanted interference as much as possible. For immediate post-surgical scanning, the operator should promptly treat mucosal bleeding and ensure intraoral drying without compromising the accuracy of the oral scan.

The original scan data were imported into EXOCAD 2.3 software (Exocad GmbH, Darmstadt, Germany), where three-dimensional reconstruction techniques were utilized to compare the differences between the oral cavity three-dimensional models obtained using different scanning rods and the traditional impression technique as the standard model. This comparison served to evaluate the scanning accuracy.

**Figure 3:**
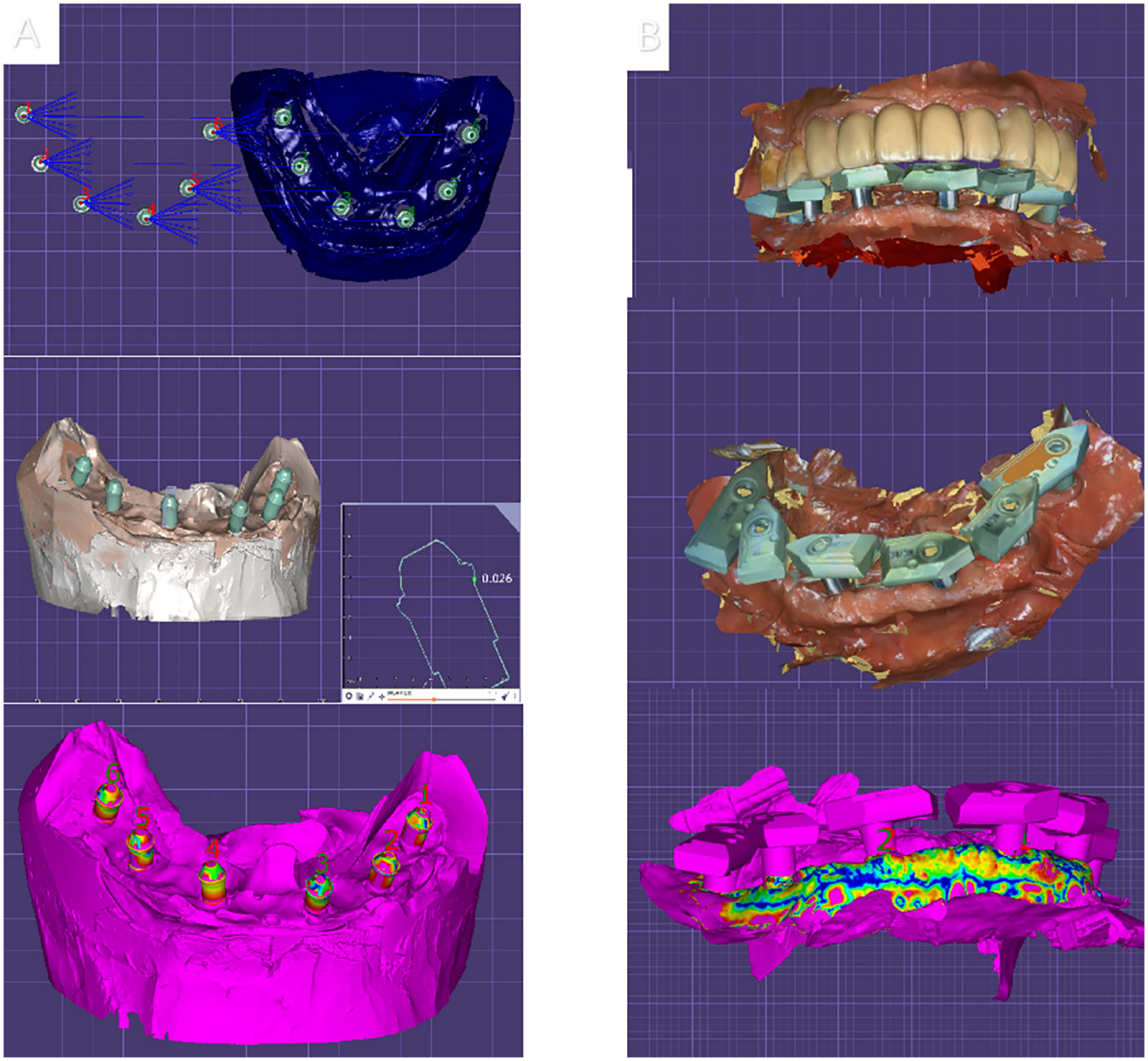
Analysis of digitized impression accuracy for two different scanning bars. **(A)** SRA original scanning bar. **(B)** Extended scanning bar.

The average error in various intraoral regions revealed a scanning accuracy of the SRA scanning rod (46 ± 45 μm), while the Segma scanning rod with an extended structure exhibited significantly higher precision (20 ± 2 μm). There is no major difference in the time required for the two scanning modalities, and a double scan of a single jaw can be accomplished in 10 min-15 min for a trained operator. In regions such as the mandibular molar area and its distal regions, where intraoral scanning is more challenging, the scanning rod with an extended structure reduced image stitching errors, thereby enhancing scanning accuracy and speed (Figure 3).

## Discussion

The Segma scanning rod with an extended structure utilized in this study incorporates advanced optical scanning technology, which enables more precise capture of implant positions and surrounding anatomical details. This results in improved accuracy and reproducibility of the digital impressions. The enhanced accuracy of the digital impressions translates into better-fitting prostheses, leading to improved patient satisfaction and long-term prognosis.

The modern dental implant concept is oriented towards restoration, requiring the implantation position of the implant to ensure the optimal functional and aesthetic effects of the prosthesis, and facilitate the long-term health and stability of the implant (10). As the implementation carrier of this concept, the implant surgical guide plays an irreplaceable bridging role in preoperative design and surgical operation. Recent studies have shown that in the process of fixed implant restoration treatment for edentulous jaws, the use of implant surgical guides is conducive to achieving the optimal position and angle of implant placement, promoting the long-term health and stability of the implant, and achieving good aesthetic results (11). By combining a radiopaque surgical guide with CBCT jaw bone information, effective communication can be achieved among surgeons, prosthodontists, and technicians in the guide design software, enabling the three-dimensional position of the implant to meet the surgeon's requirements for bone volume during surgery, the prosthodontist's requirements for the function and aesthetics of the implant restoration, and the technician's requirements for producing a high-quality restoration (12). In addition, guided implant surgery can minimize the patient's treatment costs and enable the patient to achieve better aesthetic results (13).

Oral scanning for edentulous patients offers a variety of clinical advantages, including improved impression accuracy (after taking the impression with traditional impression materials, manual filling and model trimming are required, and the tedious steps may be inaccurate), improved patient comfort (patients may experience discomfort or even adverse reactions such as nausea and vomiting when receiving traditional impressions), enhanced communication between dental professionals, and support innovative prosthodontic solutions (14). By utilizing advanced imaging technology, oral scans provide accurate digital images of the oral cavity, helping to provide more effective and personalized dental treatment for edentulous patients (15). When digital impressions are applied in edentulous patients, large image stitching errors can cause the digital scanning bar to fail to obtain accurate internal oral information (16). In this study, scanning bodies with rigid extended structures were used to increase the scanning features between adjacent implants, shorten the image stitching based on mucosal features between implants, and maximize the image registration captured by intraoral scanning based on rigid structures rather than smooth, reflective, and unstable mucosal surfaces (17). This approach aims to reduce the cumulative error generated during intraoral scanning image stitching and achieve improved scanning accuracy. The results showed that the modified scanning rod with a rigid extended structure design can improve scanning accuracy without the need for dental floss ligation in the oral cavity or additional alumina scanning marker features, thus enhancing work efficiency and patient acceptance. Additionally, the digital impression information obtained from edentulous patients receiving implant-supported fixed restoration based on the modified scanning rod facilitates clinicians, technicians, and patients to design, simulate, and communicate restoration plans. This approach is conducive to fabricating restorations that meet clinical treatment requirements while considering the patient's aesthetic needs, ultimately achieving the unification of clinical diagnosis and treatment plans, material properties, and manufacturing techniques.

However, it should be noted that the modified scanning rod is a third-party processed product. During use, it is essential to ensure correct positioning and connection between the modified scanning rod and the implant. The scanning body with an extended structure may partially obscure the mucosal information around the scanning area and implant, having a certain impact on the image fitting process (18). While there is a slight increase in material cost for the extended scan body which may increase in the overall budget for treatment. Furthermore, the existence of a learning curve for placing and scanning with the extended scan body. However, through detailed instructions and training sessions, we have found that clinicians can quickly adapt to this new technique. Finally, this study acquired the three-dimensional spatial position of the implant using reverse engineering software. This method inevitably produces inherent errors during the calculation process, which also has a certain impact on obtaining the accurate three-dimensional spatial position of the implant.

## Summary

The utilization of a Segma scanning rod with an extended structure for intraoral digital impressions in edentulous patients with implant-supported fixed restorations accurately reproduces the spatial position information of multiple implants within the oral cavity. The findings of this study provide crucial technical references for oral restoration and digital treatment, contributing to the enhancement of the quality and effectiveness of oral restoration procedures.

## Data Availability

The original contributions presented in the study are included in the article/Supplementary Material, further inquiries can be directed to the corresponding author.
